# Regarding the Influence of Additives and Additional Plasma-Induced Chemical Ionization on Adduct Formation in ESI/IMS/MS

**DOI:** 10.1021/jasms.2c00348

**Published:** 2023-04-13

**Authors:** Christian Thoben, Nora T. Hartner, Moritz Hitzemann, Christian-Robert Raddatz, Manuel Eckermann, Detlev Belder, Stefan Zimmermann

**Affiliations:** † Leibniz University Hannover, Institute of Electrical Engineering and Measurement Technology, Department of Sensors and Measurement Technology, Appelstr. 9A, 30167 Hannover, Germany; ‡ Leipzig University, Institute of Analytical Chemistry, Linnéstraße 3, 04103 Leipzig, Germany

**Keywords:** electrospray ionization, ion mobility spectrometry, IMS, IMS/MS, pesticides, additives, mobile phase, dielectric-barrier discharge, DBD, plasma source

## Abstract

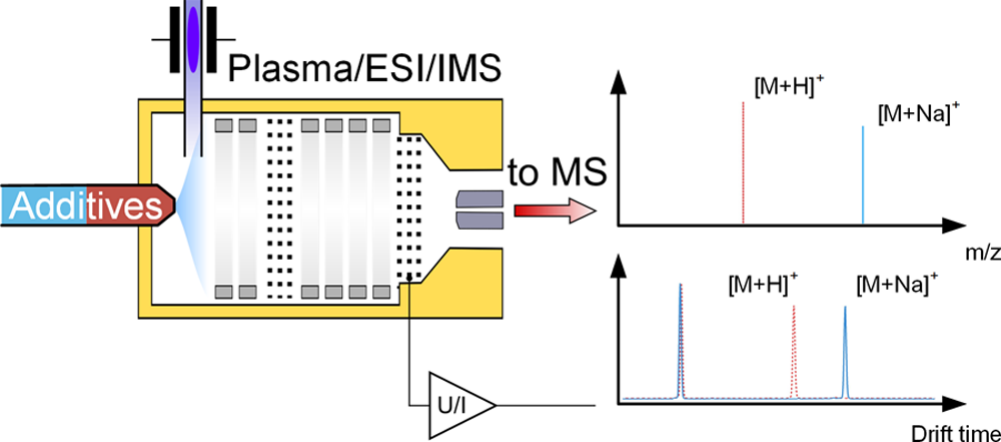

Ion mobility spectrometers (IMS) separate ions based on their ion mobility, which depends mainly on collision cross-section, mass, and charge of the ions. However, the performance is often hampered in electrospray ionization (ESI) by the appearance of multiple ion mobility peaks in the spectrum for the same analyte due to clustering and additional sodium adducts. In this work, we investigate the influence of solvents and buffer additives on the detected ion mobility peaks using ESI. Additionally, we investigate the effects of an additional chemical ionization (CI) induced by plasma ionization on the ions formed by electrospray. For this purpose, we coupled our high-resolution IMS with a resolving power of *R*_p_ = 100 to a time-of-flight mass spectrometer. Depending on the analyte and the chosen additives, the ionization process can be influenced during the electrospray process. For the herbicide isoproturon, the addition of 5 mM sodium acetate results in the formation of the sodium adduct [M + Na]^+^, which is reflected in the ion mobility *K*_0_ of 1.22 cm^2^/(V·s). In contrast, the addition of 5 mM ammonium acetate yields the protonated species [M + H]^+^ and a correspondingly higher *K*_0_ of 1.29 cm^2^/(V·s). In some cases, as with the herbicide pyrimethanil, the addition of sodium acetate can completely suppress ionizations. By carefully choosing the solvent additive for ESI-IMS or additional CI, the formation of different ion mobility peaks can be observed. This can facilitate the assignment of ions to ion mobility peaks using IMS as a compact, stand-alone instrument, e.g., for on-site analysis.

## Introduction

An electrospray is a dispersed nebula of charged droplets generated under the influence of a strong electric field. The charged droplets subsequently result in gas-phase ions. Therefore, the electrospray process is commonly used to convert liquids into the gas phase while ionizing their components. Overall, according to Cech and Enke,^1^ four possible ways to ionize analytes during the electrospray process can be assumed. The ionization type depends on the analytes and solvent. The most fundamental type of ionization is charge separation during the electrospray process. For example, ions already electrolytically dissociated in solution are separated from their counterions.^2^ Likewise, charge separation in the positive-ion mode can result in protonation of the analyte. Another option for ionization is adduct formation where in the positive-ion mode a cation transfers to the analyte molecule, similar to protonation, or an anion in the negative-ion mode, respectively. Bruins^3^ already states in his review that even analytes that are not ionizable via protonation or deprotonation due to a lack of functional groups can be ionized by adduct formation. For example, amides usually form sodium adducts. Additionally, the research group around Van Berkel^4^ investigated the electrospray ion source for its electrochemical properties. Among other things, they conclude that, in certain cases, direct electrochemical reactions can lead to ionization of analytes. According to Van Berkel et al.,^5^ this could be observed for metal–organic analytes, such as metallocenes and porphyrins, or aromatic compounds, such as polycyclic aromatic hydrocarbons.

Much discussed in the scientific community is also the ionization via gas-phase reactions. Kebarle et al.^6^ describe an ionization pathway in gas phase collisions via gas phase reactions, analogous to APCI. In this case, the gas-phase basicity of substance B must exceed that of substance A. Conversely, signal suppression can occur via gas phase reactions if the gas phase basicity of the solvent is greater than that of the analyte. In a later study by Ehrmann et al.,^7^ several substances with higher gas-phase proton affinity than the solvent methanol have been analyzed. This study showed that the observed differences in sensitivity were due to the basicity of the analytes in the liquid phase rather than their gas-phase basicity. In summary, Ehrmann et al. assumed that proton transitions by gas-phase reactions probably play a minor role in the electrospray process. However, in an ongoing debate, Yang et al.^8^ again suggest that the formation of sodium adducts and the suppression of the same by additives in the solvent are caused by gas-phase reactions.

Even though the mechanism is not yet completely understood, adduct formation is frequently observed for a variety of analytes during electrospray ionization. This complicates the interpretation of the resulting mass spectra with respect to different mass-to-charge values for the same analyte. In ion mobility spectrometry, this particular challenge is also known, leading to different drift times and ion mobilities for the protonated ion species and the sodium adduct, respectively. This again makes the ion mobility spectra difficult to interpret. In particular, the drift time differences between protonated species and sodium adduct using electrospray ionization in ion mobility spectrometry were described by Hill et al.^9^ In the positive ESI mode, the charge is usually carried by protonated solvent clusters formed by the reaction of the solvent with a weak acid (e.g., acetic, formic, or propionic acid) added to the solution. The low pH of acidic solutions offers the advantage of easier protonation of the analyte if basic functional groups are present. Furthermore, the low pH allows for constant conditions in widely used liquid chromatographic separations, which, however, will not be discussed in this work. Much more, the solvent itself has an influence on the ionization efficiencies of the analytes.^10^ The impacts of the solvent can differ in the positive and negative mode.^11,12^ However, neutral salts (e.g., ammonium acetate) are occasionally added to the solution to facilitate the analysis of polar analytes by adduct formation.

In this work, we investigate and discuss the possibility of suppressing the protonated species or the formed adducts for certain analytes by adding solvent additives. The formed ion species have been identified by their ion mobility and their mass-to-charge ratio. Therefore, we extended the previously described coupling of our compact high-resolution ambient pressure IMS to a commercial time-of-flight mass spectrometer (Bruker micrOTOF II) to include an electrospray ionization source. The IMS/MS interface used here has minimal ion losses in the transfer to the MS inlet. It allows direct measurement of ion mobility spectra and enables the gated transfer of ions into the MS to study individual ion peaks and defined drift time ranges of the ion mobility spectrum. Thus, this ESI/IMS/MS aids in the analysis and understanding of ionization processes and, ultimately, in identifying ion species formed in the ESI/IMS at ambient pressure. Moreover, a further possibility to influence the formation of adducts by an additional chemical ionization is investigated, similar to the setups for charge reduction electrospray. A combination of ESI and APCI induced by a source of corona discharge or a radioactive source is common in this field.^13−16^ In contrast, we demonstrate a way to inject reactant ions formed in a plasma source into the IMS desolvation respectively reaction region and influence ionization via electrospray by gas-phase reactions.

## Materials and Methods

### Ion Mobility Spectrometry

Separation and detection by ion mobility spectrometry, as described by Eicemann et al.,^17,18^ is based on the determination of the mobility of ions under the influence of an electric field in a gas atmosphere. In the gas phase, ions are initially accelerated in an electric field. Due to the presence of neutral atmospheric molecules, collisions occur and the ions are decelerated. The steady acceleration and the repetitive collisions result in a constant mean drift velocity, which correlates to the specific ion mobility.^19^ The ion mobility depends, among other things, on the charge of the ions, their mass, and the collision cross-section between ions and neutral particles, with the collision cross-section being the most important parameter. Based on their specific ion mobility, the different ion species can be separated within a drift gas by an electric field. The ion mobility can be described as the quotient of the square of the drift length to the product of drift voltage and drift time.

In order to correct for the influence of temperature and pressure, the reduced ion mobility is introduced, leading to a certain comparability between different setups. However, all other effects on ion mobility remain uncorrected, so that different drift gases, clustering, and even temperature can lead to different *K*_0_ values for the same compound. However, the reduced mobility *K*_0_ of all compounds is calculated according to eq 1 to
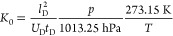
1where *l*_D_ corresponds to the length of the drift region in cm, *U*_D_ to the drift voltage, *p* to the IMS internal pressure in hPa, *T* to the temperature in the drift region in Kelvin, and *t*_D_ to the drift time of the peak in seconds.

### Instrumental

A detailed description of the used stand-alone ESI/IMS with a resolving power of *R*_p_ = 100 is given elsewhere.^20^ Ions are generated using an electrospray ion source consisting of a metal emitter (DNU-MS, New Objective Metal Taper Tip) with an inner diameter of 50 μm and a 50 mm desolvation region. To provide sample fluids to the electrospray ionization source, we use a programmable syringe pump (Advanced Microfluidics, LSPone) with a 50 μL syringe. The flow rate is set in the range from 1 to 5 μL/min. The ESI voltage is applied between the emitter and the first ring of the desolvation region. The ESI source is operated without additional sheath gas, nebulization gas, or desolvation gas.

Orthogonal to the ESI source we integrated an optional plasma ionization source. The plasma source consists of two insulated electrodes separated by a dielectric from a glass capillary located between the electrodes. By applying a high AC voltage to the electrodes, the plasma is ignited within the capillary. Further, a detailed description of the plasma ionization source is given elsewhere.^21^ The plasma source generates gas-phase reactant ions that are directed via a gas flow into the desolvation region for possible additional CI of analytes. However, such reactant ions can also affect the ions formed by ESI.

Ions are injected into the drift region via a tristate ion shutter, as already used and described in several of our IMS setups.^20,22−24^ As previously described, the interface between the IMS and MS,^25^ as well as the ion shutter, has a three-grid structure. This minimizes the influence of the potential of the center grid on the electric field in the adjacent field regions, thus avoiding field inhomogeneities.^26^ The interface serves either as a Faraday detector, recording the ion mobility spectrum at the middle grid, or as an ion shutter, transferring a certain mobility and drift time range, respectively, into the MS. The grids have a hexagonal structure with 80% optical transparency and are etched from 100 μm thick stainless steel as described before.^27^ Here, the width of the ridges between the hexagons is 80 μm.

**Figure 1:**
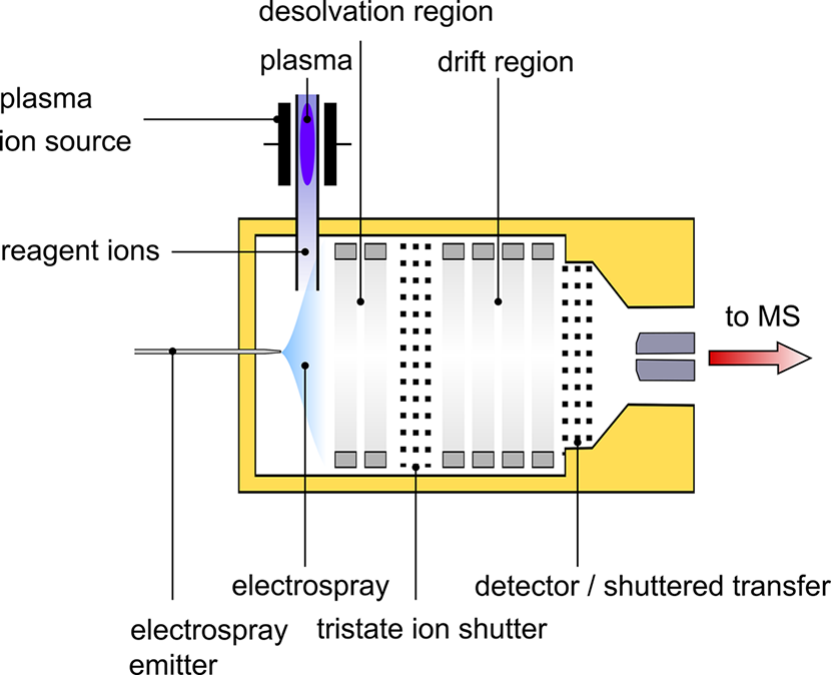
Schematic of the ESI-IMS with the electrospray emitter generating ions via electrospray ionization, the plasma source generating reactant ions in the desolvation region, and the IMS with the tristate ion shutter, which injects the generated ions into the drift region, as well as a shuttered transfer into the MS and the detection of the ion mobility spectrum, respectively.

As the potentials of the MS inlet are fixed to a certain voltage range, the detector and the amplifier, as well as the analog-to-digital conversion, are at ground potential, as in our first setup.^28^ Consequently, the inlet of the desolvation region is at high potential, and accordingly, an even higher electrical potential exists at the emitter tip. Furthermore, the IMS is supplied with a drift gas flow of 250 mL min^–1^ of purified dry air flowing in the opposite direction to the spray direction. Table 1 summarizes the relevant operating parameters. The setup is sketched in Figure 1.

**Table 1:** ESI/IMS Operating Parameters

Parameter	Value	Parameter	Value
Length of drift region	75 mm	Drift field strength	66 V/mm
Length of desolvation region	50 mm	Desolvation field strength	66 V/mm
Emitter-to-ring voltage	2–3 kV	Liquid flow to ESI	2 μL/min
Drift gas flow rate	250 mL min^–1^	Emitter diameter	50 μm
Drift gas dew point	–85 °C	Drift region temperature	25–29 °C
Drift gas	Purified dry air	Desolvation region temperature	25–29 °C
Pressure	1009–1068 hPa	Plasma excitation frequency	21 kHz
Plasma source gas flow rate	90 mL min^–1^	Plasma excitation amplitude	9 kV_peak–peak_

### Time-of-flight mass spectrometer

In this work, we use a Bruker micrOTOF II for IMS/MS coupling, as described previously.^25^ It is an atmospheric pressure ionization (API) TOF-MS with an ion flight tube containing an orthogonal acceleration stage, a reflector, and a microchannel plate detector for detection. Ions are transferred from the API source to the first vacuum stage through a glass inlet capillary reducing the pressure from ambient to 4 mbar. Skimmers, RF hexapoles, and multilens systems are used to transport the ions further through additional vacuum stages to the final pressure of 5 × 10^–7^ mbar in the ion flight tube.^29^ Our high-resolution ambient pressure ESI/IMS replaces the API source. Thus, the IMS is directly coupled to the inlet capillary of the MS. In general, the Bruker micrOTOF II is designed to measure masses up to *m*/*z* 20,000. Since this work focuses on the investigation of compounds with lower *m*/*z*, the mass range is optimized for masses from *m*/*z* 50 to 800.

### Chemicals

Cyprodinil (analytical standard, 225.29 g/mol), isoproturon (analytical standard, 206.29 g/mol), pyrimethanil (analytical standard, 199.25 g/mol), and chlortoluron (analytical standard, 212.68 g/mol) are investigated in this work. Three compositions are used as the solvent mixture, each consisting of 20% of ultrapure water (HPLC grade) and 80% of the organic solvent, either methanol (MeOH) (HPLC grade) or isopropanol (HPLC grade) or acetonitrile (ACN) (HPLC grade). Sodium acetate solution (BioUltra, 3 M), acetic acid sodium acetate buffer solution (BioUltra, 3 M), formic acid solution (BioUltra, 1 M), ammonium acetate solution (BioUltra, 5 M), and ammonium formate solution (BioUltra, 10 M) are used as additives at concentrations of 5 mM. All compounds were purchased from Sigma-Aldrich Chemie GmbH, Germany.

## Results

### ESI/IMS/MS

In a first step, the ESI/IMS/MS coupling is investigated for the analysis of the formed ion mobility peaks based on MS spectra/mass-to-charge ratios. Due to the gated transfer from the IMS to the MS, individual ion peaks from the IMS can be determined directly in the MS. In particular, this allows an exact assignment of the detected *m*/*z* values to the ion mobility peaks and a more straightforward interpretation of the results. However, it is worth noting that the ion species traveling in the IMS drift tube might differ from the ion species detected in the MS due to fragmentation, clustering, and dissociation of ions in the MS pressure stages. Since we investigate single analytes and defined ion mobility ranges the MS spectra can be interpreted accordingly.

**Figure 2:**
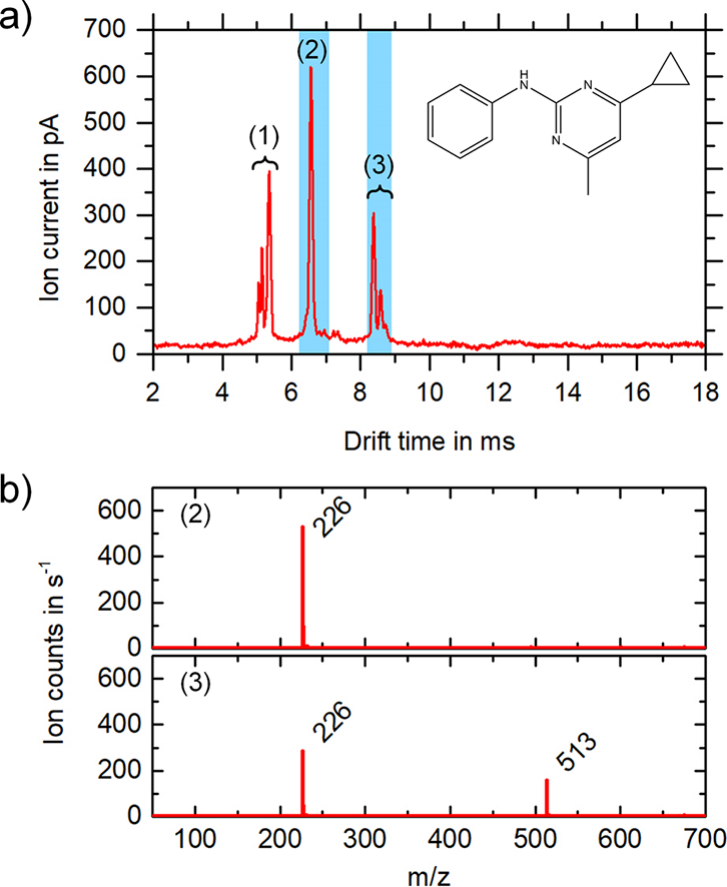
Mobility spectrum (a) and the corresponding mass spectra (b) of the individual blue marked drift time ranges of 100 mg/L cyprodinil (225 u) in 20% water and 80% acetonitrile. In the IMS, in addition to the solvent peaks (1) at *t*_D_ = 5.05 ms, *t*_D_ = 5.14 ms, and *t*_D_ = 5.35 ms and *K*_0_ = 1.71 cm^2^/(V·s), *K*_0_ = 1.68 cm^2^/(V·s), and *K*_0_ = 1.61 cm^2^/(V·s), respectively, protonated cyprodinil monomers (2) at *t*_D_ = 6.56 ms and *K*_0_ = 1.31 cm^2^/(V·s), respectively, and cyprodinil dimers (3) are measurable at *t*_D_ = 8.38 ms and *t*_D_ = 8.59 ms and *K*_0_ = 1.03 cm^2^/(V·s) and *K*_0_ = 1.00 cm^2^/(V·s), respectively. In the MS, just the protonated monomer [M + H]^+^ (*m*/*z* 226) is detected since the protonated dimer [2M + H]^+^ (*m*/*z* 451) dissociates while passing through the MS ion transfer stages. The peak at *t*_D_ = 8.59 ms and *K*_0_ = 1.03 cm^2^/(V·s), respectively, has *m*/*z* 513, which corresponds to a cluster with ACN and sodium [2M + ACN + Na]^+^.

The first substance analyzed by the ESI/IMS/MS is the fungicide cyprodinil in 20% water and 80% acetonitrile, Figure 2 shows the measured IMS and MS spectra. The typical solvent peaks (1) for ESI at *t*_D_ = 5.05 ms, *t*_D_ = 5.14 ms, and *t*_D_ = 5.35 ms and *K*_0_ = 1.71 cm^2^/(V·s), *K*_0_ = 1.68 cm^2^/(V·s), and *K*_0_ = 1.61 cm^2^/(V·s), respectively, are discriminated and not detectable with the used MS settings. The highest peak in the IMS spectra (2) at *t*_D_ = 6.56 ms and *K*_0_ = 1.31 cm^2^/(V·s), respectively, represents the protonated monomer of cyprodinil ([M + H]^+^, *m*/*z* 226), as can be clearly seen from the MS analysis in Figure 2b). For the other two peaks (3) at increased drift times of *t*_D_ = 8.38 ms and *t*_D_ = 8.59 ms and *K*_0_ = 1.03 cm^2^/(V·s) and *K*_0_ = 1.00 cm^2^/(V·s), respectively, the first of the two is most likely the protonated dimer peak ([2M + H]^+^, *m*/*z* 451), where this cluster dissociates in the MS transfer and is detected in the MS only as a protonated monomer (*m*/*z* 226). This dissociation is reasonable because reduced electric field strengths of up to 100 Td can be assumed in the transfer stage of the Bruker micrOTOF II. In addition, the pressure in the vacuum stages of the MS also supports dissociation of clusters and fragmentation.^30^ Interestingly, the peak at *t*_D_ = 8.59 ms and *K*_0_ = 1.03 cm^2^/(V·s), respectively, can be identified as a sodium adduct, being a cluster with the solvent acetonitrile ([2M + ACN + Na]^+^, *m*/*z* 513). These examples clearly show the benefit of an IMS in combination with a shuttered ion transfer into the MS for an accurate assignment of the corresponding *m*/*z* values to the ion mobility peaks and a more reliable interpretation of IMS results when used as a stand-alone device.

**Figure 3:**
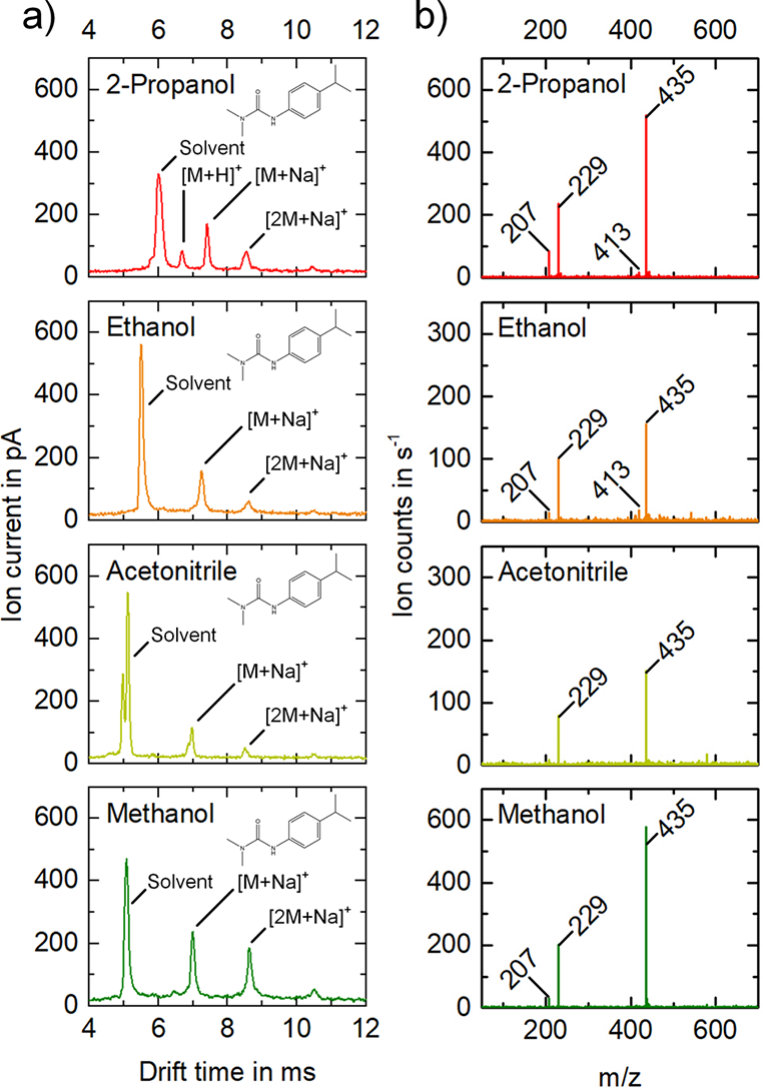
Ion mobility spectra (a) and mass spectra of the full drift time range (b) of 4 mg/L isoproturon in solvents 2-propanol (red), ethanol (orange), acetonitrile (light green), and methanol (dark green). In the IMS, protonated monomers (*m*/*z* 207), monomers as sodium adduct (*m*/*z* 229), protonated dimers (*m*/*z* 413) and dimers as sodium adduct (*m*/*z* 435) as well as trimers are formed, whereby these ion species dissociate in the MS ion transfer. With the solvent 2-propanol the solvent at *t*_D_ = 6.02 ms and *K*_0_ = 1.42 cm^2^/(V·s), respectively, protonated isoproturon monomers (*m*/*z* 207) at *t*_D_ = 6.71 ms and *K*_0_ = 1.28 cm^2^/(V·s), respectively, isoproturon monomers as sodium adduct (*m*/*z* 229) at *t*_D_ = 7.43 ms and *K*_0_ = 1.15 cm^2^/(V·s), respectively, and isoproturon dimers as sodium adduct (*m*/*z* 435) at *t*_D_ = 8.58 ms and *K*_0_ = 1.00 cm^2^/(V·s), respectively, are detectable in the ion mobility spectrum. With the solvent ethanol the solvent at *t*_D_ = 5.51 ms and *K*_0_ = 1.54 cm^2^/(V·s), respectively, isoproturon monomers as sodium adduct (*m*/*z* 229) at *t*_D_ = 7.28 ms and *K*_0_ = 1.17 cm^2^/(V·s), respectively, and isoproturon dimers as sodium adduct (*m*/*z* 435) at *t*_D_ = 8.65 ms and *K*_0_ = 0.99 cm^2^/(V·s), respectively, are measurable in the ion mobility spectrum. With the solvent acetonitrile the solvent at *t*_D_ = 4.99 ms and *t*_D_ = 5.13 ms and *K*_0_ = 1.70 cm^2^/(V·s) and *K*_0_ = 1.65 cm^2^/(V·s), respectively, isoproturon monomers as sodium adduct (*m*/*z* 229) at *t*_D_ = 7.28 ms and *K*_0_ = 1.21 cm^2^/(V·s), respectively, and isoproturon dimers as sodium adduct (*m*/*z* 435) at *t*_D_ = 8.52 ms and *K*_0_ = 0.99 cm^2^/(V·s), respectively, are detectable in the ion mobility spectrum. With the solvent methanol the solvent at *t*_D_ = 5.08 ms and *K*_0_ = 1.67 cm^2^/(V·s), respectively, isoproturon monomers as sodium adduct (*m*/*z* 229) at *t*_D_ = 7.00 ms and *K*_0_ = 1.21 cm^2^/(V·s), respectively, and isoproturon dimers as sodium adduct (*m*/*z* 435) at *t*_D_ = 8.64 ms and *K*_0_ = 0.98 cm^2^/(V·s), respectively, are measurable in the ion mobility spectrum.

In the next section, the effect of the solvent is investigated for the herbicide isoproturon. As expected, different ionization efficiencies are obtained for different solvents. The highest amplitudes for the isoproturon peaks in the ion mobility spectrum are obtained with methanol as solvent (Figure 3). Isoproturon monomers as sodium adducts ([M + Na]^+^, *m*/*z* 229) at *t*_D_ = 7.00 ms and *K*_0_ = 1.21 cm^2^/(V·s), respectively, and isoproturon dimers as sodium adducts ([2M + Na]^+^, *m*/*z* 435) at *t*_D_ = 8.64 ms and *K*_0_ = 0.98 cm^2^/(V·s), respectively, are present. Isoproturon monomers can also be detected as sodium adducts ([M + Na]^+^, *m*/*z* 229) with the solvents 2-propanol at *t*_D_ = 7.43 ms and *K*_0_ = 1.15 cm^2^/(V·s), respectively, ethanol at *t*_D_ = 7.28 ms and *K*_0_ = 1.17 cm^2^/(V·s), respectively, and acetonitrile at *t*_D_ = 7.28 ms and *K*_0_ = 1.21 cm^2^/(V·s), respectively. In addition, isoproturon dimers can also be identified as sodium adducts ([2M + Na]^+^, *m*/*z* 435) with 2-propanol at *t*_D_ = 8.58 ms and *K*_0_ = 1.00 cm^2^/(V·s), respectively, ethanol at *t*_D_ = 8.65 ms and *K*_0_ = 0.99 cm^2^/(V·s), respectively, and acetonitrile at *t*_D_ = 8.52 ms and *K*_0_ = 0.99 cm^2^/(V·s), respectively. In contrast to the previous measurement with cyprodinil, an increased sodium adduct formation can be observed with isoproturon, which in comparison has a diamide group. However, the highest value for the protonated monomer of isoproturon ([M + H]^+^, *m*/*z* 207) is reached with 2-propanol as solvent. Here, the protonated monomer can be seen in the IMS spectrum at *t*_D_ = 6.71 ms and *K*_0_ = 1.28 cm^2^/(V·s), respectively, which is not the case with the other solvents. These experiments clearly show that even though each peak can be assigned based on the *m*/*z* ratio, the occurrence of adducts and multiple ion mobility peaks for one analyte complicates interpretation of IMS results when used as a stand-alone device.

**Figure 4:**
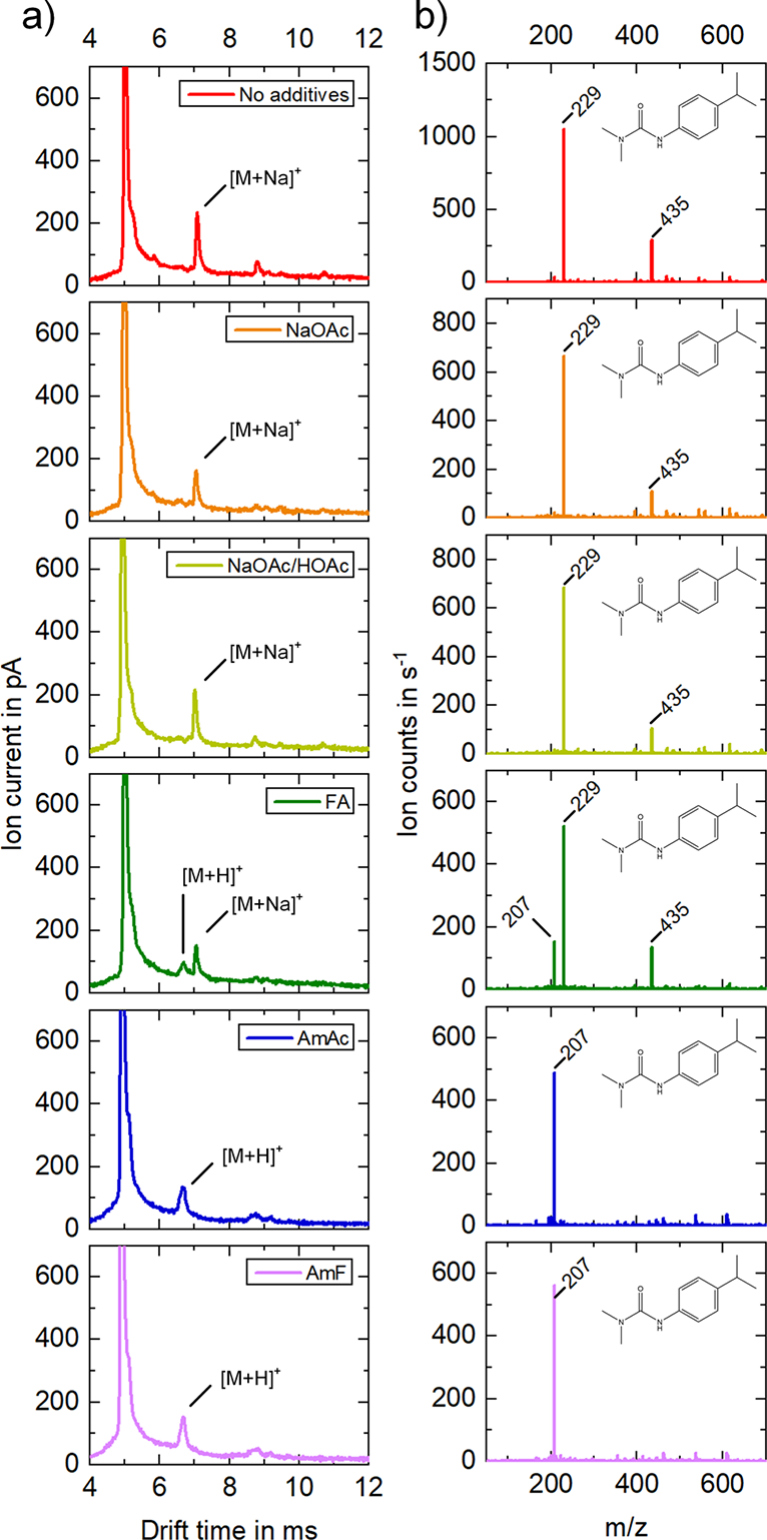
Ion mobility spectra (a) and mass spectra of the full drift time range (b) of 4 mg/L isoproturon in 20% water and 80% methanol for comparison without additives (red) and with single additives (5 mM): sodium acetate (orange), acetic acid sodium acetate buffer (light green), formic acid (dark green), ammonium acetate (blue), and ammonium formate (violet). With no additives, isoproturon monomers as sodium adduct (*m*/*z* 229) at *t*_D_ = 7.08 ms and *K*_0_ = 1.21 cm^2^/(V·s), respectively, are detectable. With the addition of 5 mM sodium acetate isoproturon monomers as sodium adduct (*m*/*z* 229) at *t*_D_ = 7.05 ms and *K*_0_ = 1.22 cm^2^/(V·s), respectively, are detectable. With the addition of 5 mM acetic acid sodium acetate buffer isoproturon monomers as sodium adduct (*m*/*z* 229) at *t*_D_ = 7.02 ms and *K*_0_ = 1.22 cm^2^/(V·s), respectively, are measurable. With the addition of 5 mM formic acid protonated isoproturon monomers (*m*/*z* 207) at *t*_D_ = 6.69 ms and *K*_0_ = 1.29 cm^2^/(V·s), respectively, and isoproturon monomers as sodium adduct (*m*/*z* 229) at *t*_D_ = 7.04 ms and *K*_0_ = 1.22 cm^2^/(V·s), respectively, are detectable. With the addition of 5 mM ammonium acetate protonated isoproturon monomers (*m*/*z* 207) at *t*_D_ = 6.69 ms and *K*_0_ = 1.29 cm^2^/(V·s), respectively, are measurable. With the addition of 5 mM ammonium formate protonated isoproturon monomers (*m*/*z* 207) at *t*_D_ = 6.67 ms and *K*_0_ = 1.28 cm^2^/(V·s), respectively, are detectable.

In order to investigate the influence of additives on the formation of protonated ion species and sodium adducts in the electrospray process, various additives were added to the analyte solution (Figure 4). As can be seen from Figure 4, either the sodium adduct or the protonated species or both are formed when using isoproturon as analyte. In the case of sodium acetate at *t*_D_ = 7.05 ms and *K*_0_ = 1.22 cm^2^/(V·s), respectively, and acetic acid sodium acetate buffer at *t*_D_ = 7.02 ms and *K*_0_ = 1.22 cm^2^/(V·s), respectively, only sodium adducts ([M + Na]^+^, *m*/*z* 229) are observed, as expected. In contrast, the addition of ammonium-based buffers (ammonium acetate and formate) results in the formation of only one ion mobility peak, which corresponds to the protonated ion ([M + H]^+^, *m*/*z* 207). The protonated monomer can be measured in the ion mobility spectrum in the case of the additive ammonium acetate at *t*_D_ = 6.69 ms and *K*_0_ = 1.29 cm^2^/(V·s), respectively, and in the case of the additive ammonium formate at *t*_D_ = 6.67 ms and *K*_0_ = 1.28 cm^2^/(V·s), respectively. When formic acid is added, both the protonated isoproturon monomers ([M + H]^+^, *m*/*z* 207) at *t*_D_ = 6.69 ms and *K*_0_ = 1.29 cm^2^/(V·s), respectively, and sodiated isoproturon monomers ([M + Na]^+^, *m*/*z* 229) at *t*_D_ = 7.04 ms and *K*_0_ = 1.22 cm^2^/(V·s), respectively, are observed. In addition, we repeated this experiment with acetic acid and obtained the same results showing protonated monomers and sodiated monomers. For comparison, measurements were carried out without additives, in which isoproturon tends to form sodium adducts at *t*_D_ = 7.08 ms and *K*_0_ = 1.21 cm^2^/(V·s), respectively.

**Figure 5:**
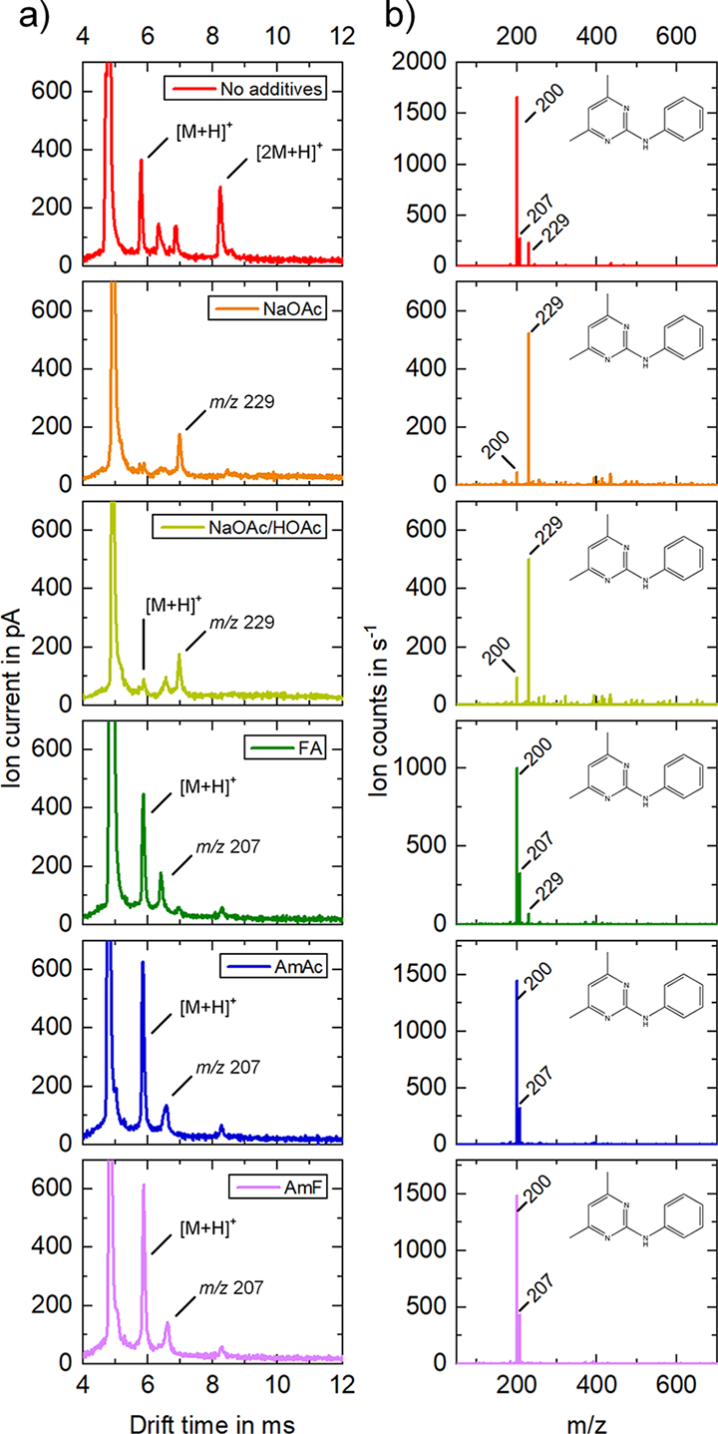
Ion mobility spectra (a) and mass spectra of the full drift time range (b) of 4 mg/L pyrimethanil in 20% water and 80% methanol for comparison without additives (red) and with single additives (5 mM): sodium acetate (orange), acetic acid sodium acetate buffer (light green), formic acid (dark green), ammonium acetate (blue), and ammonium formate (violet). With no additives protonated pyrimethanil monomers (*m*/*z* 200) at *t*_D_ = 5.80 ms and *K*_0_ = 1.41 cm^2^/(V·s), respectively, and protonated pyrimethanil dimers (*m*/*z* 399) at *t*_D_ = 8.24 ms and *K*_0_ = 1.00 cm^2^/(V·s), respectively, are detectable, as well as impurities of protonated isoproturon monomers (*m*/*z* 207) at *t*_D_ = 6.34 ms and *K*_0_ = 1.29 cm^2^/(V·s), respectively, and isoproturon monomers as sodium adduct (*m*/*z* 229) at *t*_D_ = 6.88 ms and *K*_0_ = 1.19 cm^2^/(V·s), respectively. With the addition of 5 mM sodium acetate predominantly impurities of isoproturon monomers as sodium adduct (*m*/*z* 229) at *t*_D_ = 6.99 ms and *K*_0_ = 1.17 cm^2^/(V·s), respectively, are measurable. With the addition of 5 mM acetic acid sodium acetate buffer a small among of protonated pyrimethanil monomers (*m*/*z* 200) at *t*_D_ = 5.88 ms and *K*_0_ = 1.40 cm^2^/(V·s), respectively, and predominantly impurities of isoproturon monomers as sodium adduct (*m*/*z* 229) at *t*_D_ = 6.98 ms and *K*_0_ = 1.17 cm^2^/(V·s), respectively, are detectable. With the addition of 5 mM formic acid protonated pyrimethanil monomers (*m*/*z* 200) at *t*_D_ = 5.87 ms and *K*_0_ = 1.39 cm^2^/(V·s), respectively, and impurities of protonated isoproturon monomers (*m*/*z* 207) at *t*_D_ = 6.41 ms and *K*_0_ = 1.28 cm^2^/(V·s), respectively, are measurable. With the addition of 5 mM ammonium acetate protonated pyrimethanil monomers (*m*/*z* 200) at *t*_D_ = 5.85 ms and *K*_0_ = 1.40 cm^2^/(V·s), respectively, and impurities of protonated isoproturon monomers (*m*/*z* 207) at *t*_D_ = 6.57 ms and *K*_0_ = 1.25 cm^2^/(V·s), respectively, are detectable. With the addition of 5 mM ammonium formate protonated pyrimethanil monomers (*m*/*z* 200) at *t*_D_ = 5.88 ms and *K*_0_ = 1.39 cm^2^/(V·s), respectively, and impurities of protonated isoproturon monomers (*m*/*z* 207) at *t*_D_ = 6.61 ms and *K*_0_ = 1.24 cm^2^/(V·s), respectively, are measurable.

If pyrimethanil, which only forms sodium adducts to a limited extent, is examined in an analogous fashion, the protonated pyrimethanil (*m*/*z* 200) peaks were suppressed in the IMS spectrum by adding sodium acetate (Figure 5). This suppression effect also occurs with the additive acetic acid sodium acetate buffer, to a smaller degree. For all other additives added to the pyrimethanil solution, this suppression effect does not exist. In the case of sodium acetate as additive, only residues of isoproturon from the previous measurement were detected as sodium adducts at *t*_D_ = 6.99 ms and *K*_0_ = 1.17 cm^2^/(V·s), respectively. However, with the addition of 5 mM acetic acid sodium acetate buffer protonated pyrimethanil monomers ([M + H]^+^, *m*/*z* 200) are observed to a small extent at *t*_D_ = 5.88 ms and *K*_0_ = 1.40 cm^2^/(V·s), respectively, and predominantly impurities of isoproturon monomers as sodium adduct (*m*/*z* 229) are observed at *t*_D_ = 6.98 ms and *K*_0_ = 1.17 cm^2^/(V·s), respectively. As expected, with the additive of 5 mM formic acid protonated pyrimethanil monomers ([M + H]^+^, *m*/*z* 200) at *t*_D_ = 5.87 ms and *K*_0_ = 1.39 cm^2^/(V·s), respectively, and impurities of protonated isoproturon monomers (*m*/*z* 207) at *t*_D_ = 6.41 ms and *K*_0_ = 1.28 cm^2^/(V·s), respectively, are detected. In addition, we repeated this experiment with acetic acid and again obtained the same results, mainly showing protonated monomers. With the addition of 5 mM ammonium acetate likewise protonated pyrimethanil monomers ([M + H]^+^, *m*/*z* 200) at *t*_D_ = 5.85 ms and *K*_0_ = 1.40 cm^2^/(V·s), respectively, and impurities of protonated isoproturon monomers (*m*/*z* 207) at *t*_D_ = 6.57 ms and *K*_0_ = 1.25 cm^2^/(V·s), respectively, are present. With the addition of 5 mM ammonium formate again protonated pyrimethanil monomers ([M + H]^+^, *m*/*z* 200) at *t*_D_ = 5.88 ms and *K*_0_ = 1.39 cm^2^/(V·s), respectively, and impurities of protonated isoproturon monomers (*m*/*z* 207) at *t*_D_ = 6.61 ms and *K*_0_ = 1.24 cm^2^/(V·s), respectively, are measurable.

**Figure 6:**
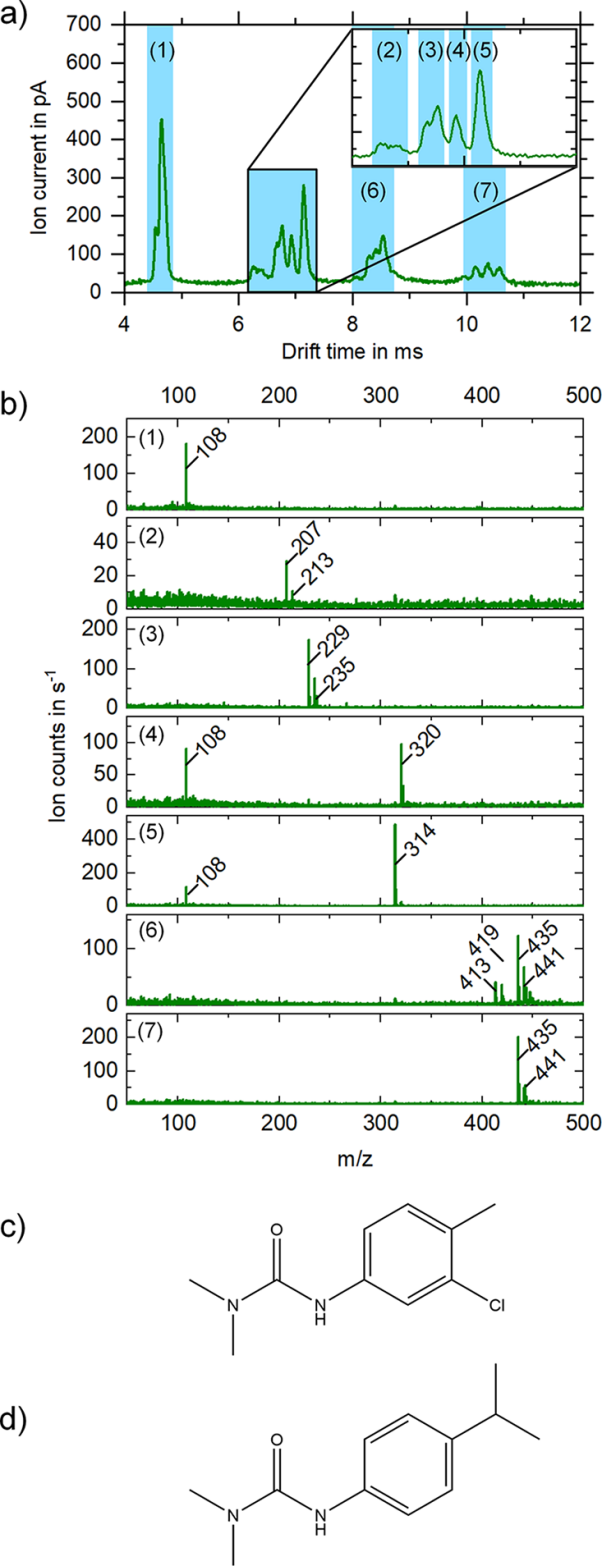
Ion mobility spectrum (a) and the corresponding mass spectra (b) of the individual blue marked ion mobility sections of the mixture of 10 mg/L chlortoluron (212 u) and 10 mg/L isoproturon (206 u) in 20% water and 80% methanol. In addition to the solvent peaks (1) at *t*_D_ = 4.55 ms and *t*_D_ = 4.65 ms and *K*_0_ = 1.88 cm^2^/(V·s) and *K*_0_ = 1.84 cm^2^/(V·s), respectively, protonated isoproturon monomers (*m*/*z* 207) at *t*_D_ = 6.26 ms and *K*_0_ = 1.37 cm^2^/(V·s), respectively, and protonated chlortoluron monomers (*m*/*z* 213) at *t*_D_ = 6.40 ms and *K*_0_ = 1.34 cm^2^/(V·s), respectively, (2) and isoproturon monomers as sodium adducts (*m*/*z* 229) at *t*_D_ = 6.67 ms and *K*_0_ = 1.28 cm^2^/(V·s), respectively, and chlortoluron monomers as sodium adducts (*m*/*z* 235) at *t*_D_ = 6.77 ms and *K*_0_ = 1.26 cm^2^/(V·s), respectively, (3) as well as chlortoluron solvent cluster (*m*/*z* 320) at *t*_D_ = 6.93 ms and *K*_0_ = 1.24 cm^2^/(V·s), respectively, (4) isoproturon solvent cluster (*m*/*z* 314) at *t*_D_ = 7.14 ms and *K*_0_ = 1.20 cm^2^/(V·s), respectively, (5) and dimers in the drift time range of *t*_D_ = 8.07–8.54 ms and the ion mobility range of *K*_0_ = 1.06–1.00 cm^2^/(V·s), respectively, (6) and trimers in the drift time range of *t*_D_ = 10.15–10.58 ms and the ion mobility range of *K*_0_ = 0.84–0.81 cm^2^/(V·s), respectively, (7) are measurable in the IMS. The structural formula for chlortoluron is shown in (c) and the one for isoproturon in (d).

Finally, a mixture of isoproturon and chlortoluron with and without additives was investigated to determine the effect of additives on more complex IMS spectra. As can be seen in Figure 6a, three groups of ion peaks (*t*_D_ = 6.40–7.14 ms, *t*_D_ = 8.07–8.54 ms, and *t*_D_ = 10.15–10.58 ms) were formed in addition to the solvent peaks at *t*_D_ = 4.55 ms and *t*_D_ = 4.65 ms and *K*_0_ = 1.88 cm^2^/(V·s) and *K*_0_ = 1.84 cm^2^/(V·s), respectively. Likewise, a corresponding number of mass-to-charge ratios were detected in the mass spectrometer. As before, a defined drift time range of the ion mobility spectrum is mass analyzed and thus allows easy identification of the underlying ion species of certain peaks in the ion mobility spectrum. Interestingly, solvent clusters were formed for chlortoluron ([M_C_ + S]^+^, *m*/*z* 320) at *t*_D_ = 6.93 ms and *K*_0_ = 1.24 cm^2^/(V·s), respectively, as well as isoproturon ([M_I_ + S]^+^, *m*/*z* 314) at *t*_D_ = 7.14 ms and *K*_0_ = 1.20 cm^2^/(V·s), respectively. These clusters have lower ion mobilities than the singly charged monomers due to their higher mass and collision cross-section and, therefore, longer drift time. As in the previous measurements, protonated species and sodium adducts can be identified for the corresponding monomers and dimers. The protonated isoproturon monomers ([M_I_ + H]^+^, *m*/*z* 207) exhibit a drift time of *t*_D_ = 6.26 ms and *K*_0_ = 1.37 cm^2^/(V·s), respectively, and the protonated chlortoluron monomers ([M_C_ + H]^+^, *m*/*z* 213) exhibit a drift time of *t*_D_ = 6.40 ms and *K*_0_ = 1.34 cm^2^/(V·s), respectively. In Addition, the isoproturon monomers as sodium adducts ([M_I_ + Na]^+^, *m*/*z* 229) exhibit a drift time of *t*_D_ = 6.67 ms and *K*_0_ = 1.28 cm^2^/(V·s), respectively, and the chlortoluron monomers as sodium adducts ([M_C_ + Na]^+^, *m*/*z* 235) exhibit a drift time of *t*_D_ = 6.77 ms and *K*_0_ = 1.26 cm^2^/(V·s), respectively This also explains the large number of peaks in the ion mobility spectrum, including dimers in the drift time range of *t*_D_ = 8.07–8.54 ms and the ion mobility range of *K*_0_ = 1.06–1.00 cm^2^/(V·s), respectively, and trimers in the drift time range of *t*_D_ = 10.15–10.58 ms and the ion mobility range of *K*_0_ = 0.84–0.81 cm^2^/(V·s), respectively. The MS data of the analyzed peaks are shown in Figure 6 andTable 2 summarizes the identified peaks.

**Table 2:** Mass-to-Charge Ratios and Ion Species for 10 mg/L Chlortoluron (212 u) and 10 mg/L Isoproturon (206 u) in 20% Water and 80% Methanol

*m*/*z*	Ion species	Description
108	S^+^	Solvent peak
207	[M_I_ + H]^+^	Protonated isoproturon
213	[M_C_ + H]^+^	Protonated chlortoluron
229	[M_I_ + Na]^+^	Isoproturon as sodium adduct
235	[M_C_ + Na]^+^	Chlortoluron as sodium adduct
314	[M_I_ + S]^+^	Isoproturon solvent cluster
320	[M_C_ + S]^+^	Chlortoluron solvent cluster
413	[2M_I_ + H]^+^	Protonated isoproturon dimer
419	[M_I_ + M_C_ + H]^+^	Mixed protonated dimer
435	[2M_I_ + Na]^+^	Isoproturon dimer as sodium adduct
441	[M_I_ + M_C_ + Na]^+^	Mixed dimer as sodium adduct

**Figure 7:**
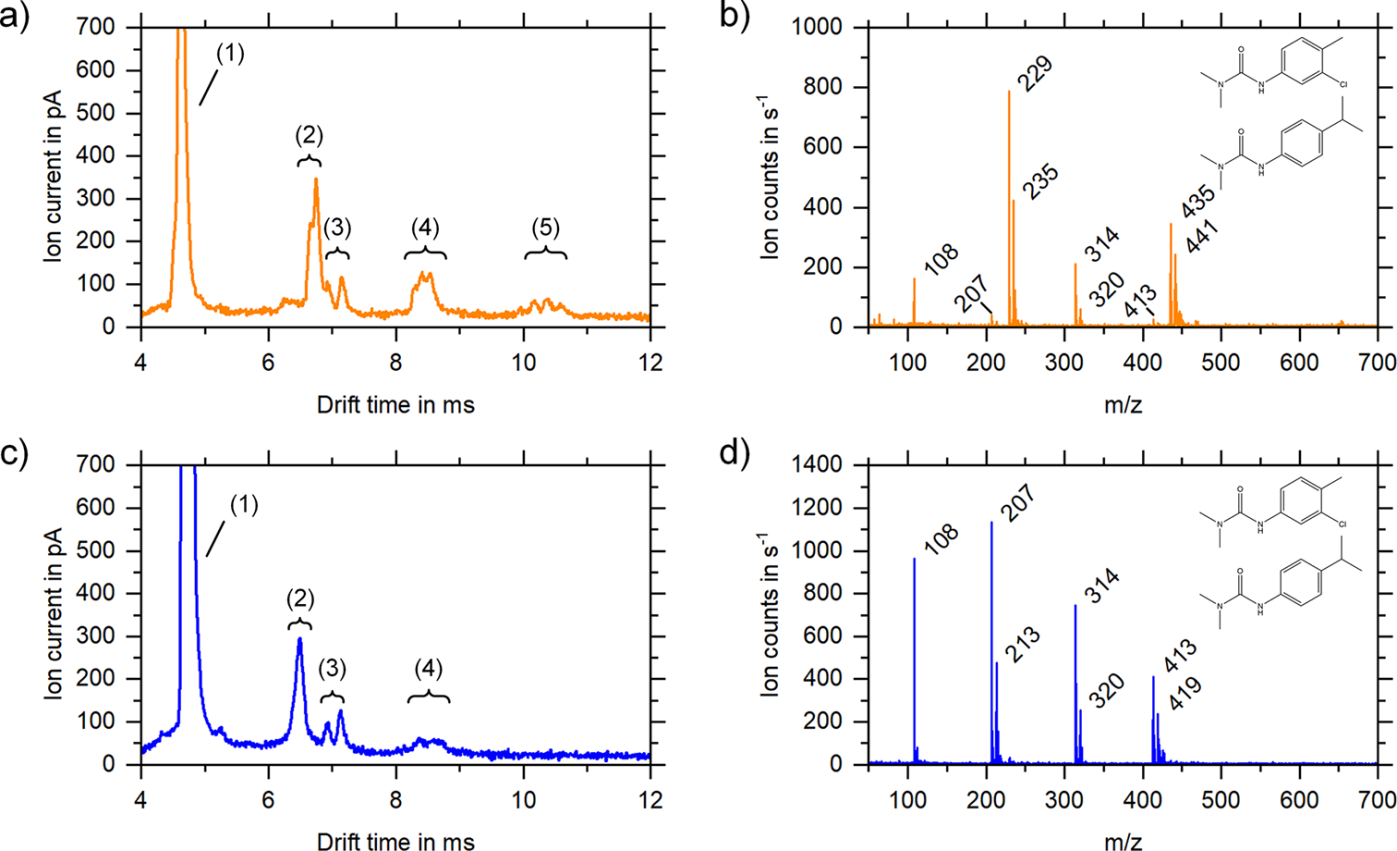
Ion mobility spectra (a) and (c) and mass spectra (b) and (d) of the mixture of 10 mg/L chlortoluron (212 u) and 10 mg/L isoproturon (206 u) with 5 mM sodium acetate (a) and (b) and 5 mM ammonium acetate (c) and (d), respectively, in 20% water and 80% methanol. With 5 mM sodium acetate, monomers (2) in the drift time range of *t*_D_ = 6.65–6.74 ms and the ion mobility range of *K*_0_ = 1.28–1.26 cm^2^/(V·s), respectively, dimers (4) in the drift time range of *t*_D_ = 8.31–8.54 ms and the ion mobility range of *K*_0_ = 1.03–1.00 cm^2^/(V·s), respectively, and trimers (5) in the drift time range of *t*_D_ = 10.18–10.58 ms and the ion mobility range of *K*_0_ = 0.84–0.81 cm^2^/(V·s), respectively, as well as cluster compounds with the solvent (3) in the drift time range of *t*_D_ = 6.92–7.15 ms and the ion mobility range of *K*_0_ = 1.19–1.23 cm^2^/(V·s), respectively, are measurable in the IMS in addition to the solvent peak (1). With 5 mM ammonium acetate, monomers (2) in the drift time range of *t*_D_ = 6.40–6.48 ms and the ion mobility range of *K*_0_ = 1.33–1.31 cm^2^/(V·s), respectively, dimers (4) in the drift time range of *t*_D_ = 8.37–8.67 ms and the ion mobility range of *K*_0_ = 1.02–0.98 cm^2^/(V·s), respectively, as well as cluster compounds with the solvent (3) in the drift time range of *t*_D_ = 6.93–7.13 ms and the ion mobility range of *K*_0_ = 1.20–1.23 cm^2^/(V·s), respectively, are measurable in the IMS in addition to the solvent peak (1).

If 5 mM sodium acetate is added to the mixture, sodium adducts of the monomers are increasingly formed. This is also reflected in the larger amplitudes of the peaks in the ion mobility spectrum compared to the previous measurement without addition, such as the monomers as sodium adducts ([M + Na]^+^, *m*/*z* 229 and *m*/*z* 235) in the drift time range of *t*_D_ = 6.65–6.74 ms and the ion mobility range of *K*_0_ = 1.28–1.26 cm^2^/(V·s), respectively. However, the protonated species (in particular the protonated isoproturon *m*/*z* 207 and 413), are still detectable in the IMS and MS, indicated in Figure 7a,b. The corresponding ion species are given in Table 2. Besides the monomers, their solvent clusters with *m*/*z* 314 and *m*/*z* 320 in the drift time range of *t*_D_ = 6.93–7.13 ms and the ion mobility range of *K*_0_ = 1.20–1.23 cm^2^/(V·s), respectively, and the dimers as sodium adducts with *m*/*z* 435 and *m*/*z* 441 in the drift time range of *t*_D_ = 8.31–8.54 ms and the ion mobility range of *K*_0_ = 1.03–1.00 cm^2^/(V·s), respectively, are again detectable. In the IMS, also trimers in the drift time range of *t*_D_ = 10.18–10.58 ms and the ion mobility range of *K*_0_ = 0.84–0.81 cm^2^/(V·s), respectively, are also present.

However, adding 5 mM of ammonium acetate suppresses all sodium adducts. In the IMS/MS setup, only the protonated isoproturon ([M_I_ + H]^+^, *m*/*z* 207) and chlortoluron ([M_C_ + H]^+^, *m*/*z* 213) are detected in the drift time range of *t*_D_ = 6.40–6.48 ms and the ion mobility range of *K*_0_ = 1.33–1.31 cm^2^/(V·s), respectively, and their solvent clusters ([M_I_ + S]^+^, *m*/*z* 314 and ([M_C_ + S]^+^, *m*/*z* 320) in the drift time range of *t*_D_ = 6.93–7.13 ms and the ion mobility range of *K*_0_ = 1.20–1.23 cm^2^/(V·s), respectively, as well as protonated dimers, *m*/*z* 413 and *m*/*z* 419, in the drift time range of *t*_D_ = 8.37–8.67 ms and the ion mobility range of *K*_0_ = 1.02–0.98 cm^2^/(V·s), respectively. In addition, the trimers are no longer detected with the IMS. This simplifies the assignment of the peaks in the ion mobility spectrum to the corresponding ion species, and it solves the issue that both ion species, protonated [M + H]^+^ and sodium-bound monomer [M + Na]^+^ and beyond that their multimers, have to be observed and thus separated in the IMS. This is particularly useful when using an IMS as a stand-alone device, for example, if the instrumental effort or the size of the instrument in a certain application does not allow for ESI/IMS/MS or ESI/MS. However, the protonated species of isoproturon ([M_I_ + H]^+^, *m*/*z* 207) and chlortoluron ([M_C_ + H]^+^, *m*/*z* 213) cannot be separated with the available IMS. The corresponding measurements are shown in Figure 7c,d, and the ion species and mass-to-charge ratios are shown in Table 2.

### Additional CI via Reactant Ions from a Plasma source

Another possibility to influence the formation of adducts is the use of an additional CI via reactant ions formed in a plasma source and fed into the IMS desolvation/reaction region. Often found in this context is a combination of ESI and APCI induced by a corona discharge^31−33^ or setups with a fast switching dual (ESI/APCI) ionization source.^34^ Dual sources with atmospheric pressure photoionization (APPI)^35^ and coaxial plasma ionization sources^36^ have also been described. Here, we use a plasma ionization source (DBD) that generates reactant ions that are fed with a gas flow into the IMS desolvation respectively reaction region after the ESI emitter. This avoids field distortion in the IMS desolvation respectively reaction region, as it would be the case for an orthogonally mounted corona discharge needle. Another advantage of the additional source is that it can be easily switched on and off. The reactant ions coming from the plasma source are reactive ions or excited species generated within or downstream of the plasma. The plasma gas used is nitrogen with a sufficient residual amount of water. Within the plasma, the nitrogen is directly ionized by electron impact.^37^ The ionized nitrogen, in turn, can generate protonated water clusters in a reaction cascade.^38−40^ Subsequently, these ions enter the ionization region of the IMS via a gas flow. Here, the additionally introduced ions influence the electrospray process or the reactant ions can ionize the analytes by chemical or Penning ionization.

**Figure 8:**
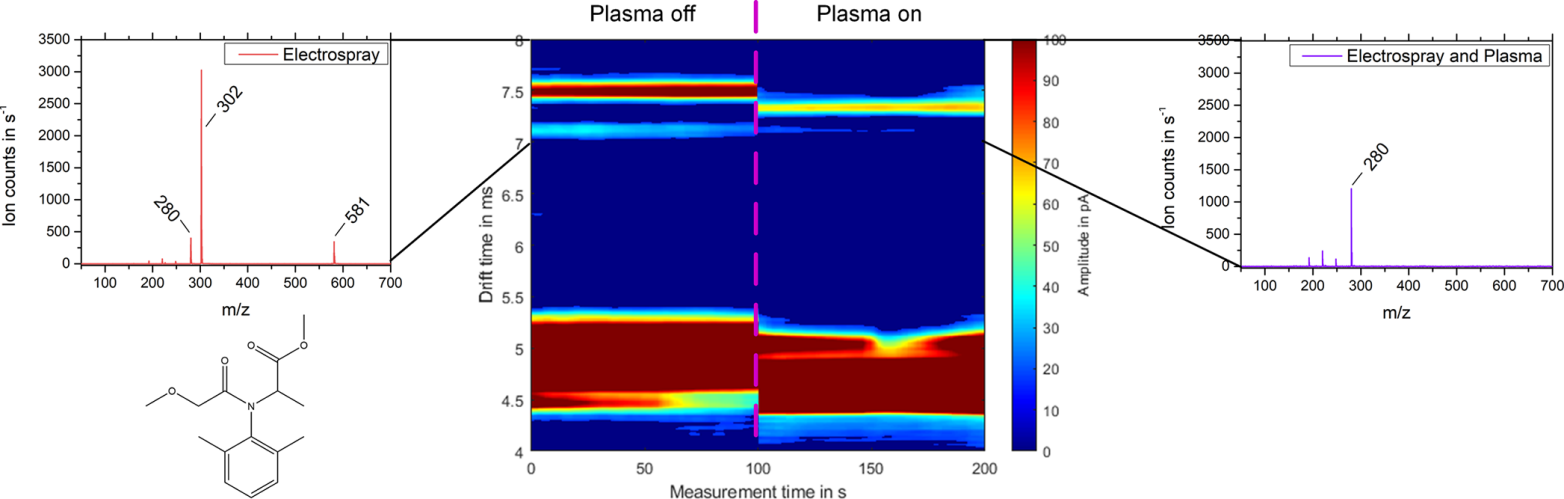
2D plot of the ion mobility spectrum of 1 mg/L metalaxyl in 70:30 ACN:H_2_O versus measuring time, with drift time plotted on the ordinate and amplitude coded across the color scale. On the left side only the electrospray source is operated; on the right side the plasma source is turned on after 100 s measuring time. The corresponding mass spectra show the drift time section between 7 and 8 ms. When the plasma source is turned off, a major peak at *t*_D_ = 7.49 ms and *K*_0_ = 1.15 cm^2^/(V·s), respectively, is measurable related to the monomers as sodium adduct [M + Na]^+^ (*m*/*z* 302). In addition, a smaller second peak at *t*_D_ = 7.11 ms and *K*_0_ = 1.21 cm^2^/(V·s), respectively, exists. When the plasma source is turned on, a principal peak at *t*_D_ = 7.34 ms and *K*_0_ = 1.17 cm^2^/(V·s), respectively, is measurable related to the protonated monomer [M + H]^+^ (*m*/*z* 280). The mass spectrum also obtained the transient signals of metalaxyl fragmentation at *m*/*z* 248, *m*/*z* 220, and *m*/*z* 192.

Figure 8 shows a very first measurement with this setup and the fungicide metalaxyl as analyte. Initially, the system is only in ESI mode and the metalaxyl forms a sodium adduct [M + Na]^+^ at *t*_D_ = 7.49 ms and *K*_0_ = 1.15 cm^2^/(V·s), respectively, which is also detected in the MS with *m*/*z* 302. Besides, the mass spectrum also obtained 280 and *m*/*z* 581 associated with the protonated monomer [M + H]^+^ and the sodium-bonded dimer [2 M + Na]^+^, respectively. After 100 s of measurement time, the plasma source is turned on and the formation of a different ion species is clearly visible. The drift time in the IMS changes to *t*_D_ = 7.11 ms and *K*_0_ = 1.21 cm^2^/(V·s), respectively, and in the MS, we now see predominantly the protonated metalaxyl monomer [M + H]^+^ with *m*/*z* 280. The mass spectrum also obtained the well-known transient signals of metalaxyl fragmentation at *m*/*z* 248, 220, and 192.

## Discussion

It is a well-known phenomenon that electrospray ionization leads to the formation of protonated ions and adduct formation, especially with sodium.^1,41^ Due to different masses and collision cross sections, differences in drift time and ion mobility, respectively, could be observed for the different ion species in previous ESI/IMS/MS studies.^42,43^ Shibue et al.^44^ have investigated the formation of sodium adducts with ESI/MS. In their work, molecules that have nitrogen but no oxygen in their elemental composition are mainly protonated. Furthermore, they introduce the topological polar surface area (tPSA) to estimate the ion species formed of molecules. They deduce that molecules with a small tPSA less than or equal to 30 Å^2^ and a relatively low molecular weight below 250 u are more likely to be purely protonated. With increasing tPSA and larger molecular weight, sodium adducts occur more predominantly. This is also consistent with our results. However, the tPSA value alone does not allow any prediction about the expected ionization mode for the analytes studied here. Rather, it is the presence of oxygen in the molecule that increases the probability of sodium adduct formation. The corresponding tPSA and molecular weight values of the analytes studied here are listed in

Table 3. As expected, cyprodinil forms a protonated monomer. Interestingly, cluster formation with the solvent acetonitrile results in the formation of a sodium adduct; see Figure 2. Provided we consider the cluster as a unit, it can be assumed that the acetonitrile increases the tPSA to 55.3 Å^2^, thus favoring sodium adduct formation. Furthermore, we could show that adduct formation cannot be forced by additives if the molecule does not have oxygen. An interesting discovery is that pyrimethanil is actually completely suppressed upon the addition of sodium acetate; see Figure 5.

**Table 3:** Molecular Formula, Molecular Weight, and tPSA

Analyte	Molecular formula	Molecular weight	tPSA in Å^2^
Cyprodinil	C_14_H_15_N_3_	225 u	35.4
Isoproturon	C_12_H_18_N_2_O	206 u	37.8
Pyrimethanil	C_12_H_13_N_3_	199 u	35.4
Chlortoluron	C_10_H_13_ClN_2_O	212 u	37.8
Metalaxyl	C_15_H_21_NO_4_	279 u	75.5

The studies by Wong et al.^8^ show suppression of sodium adducts starting at low additive concentration of 0.4 mM ammonia. Similarly, the addition of ammonium acetate also leads to suppression of sodium adducts as shown in Figures 4 and 5. Mortier et al.^45^ also show similar results with paclitaxel, such as the reduction of adducts when using ammonium format and an increase in sodium adduct intensity with sodium acetate while suppressing the protonated analyte.

In addition, only sodium adducts were generally detected in our study if oxygen is present in the molecular structure of the analyte. In contrast, Kruve et al.^46^ do observe the formation of a sodium adduct with 1,10-phenanthroline (tPSA: 22.6 Å^2^; 180 u) even in the absence of oxygen, but at a much higher sodium acetate concentration of 100 mM. The experimental data presented in Figure 3 are also consistent with the fact that when acetonitrile is used as a solvent, mainly sodium adducts are formed, similar to the described results in another study by Kruve et al.^47^ However, lower intensities are measured in the IMS and MS with acetonitrile compared to, e.g., methanol. This also matches the results of Rebane et al.^48^ Furthermore, corresponding to the work of Kruve et al. on the formation efficiency of protonated molecules and sodium adducts,^47^ all analytes are readily ionizable even without additives and therefore show high signal intensities. In agreement with the investigations of Kruve et al., the formation of sodium adducts could not only be reduced but also completely suppressed by the addition of ammonium acetate.

A possible explanation for the reduction and even suppression of sodium adducts might be found in the different surface affinities within the primary droplets during the electrospray process. Various research groups have described the enrichment of H^+^ and H_3_O^+^, respectively, at the surface of water–air interfaces,^49−51^ but not for Na^+^. Due to the dissociation of ammonium, H_3_O^+^ is also formed with the addition of ammonium acetate in the analyte solution. Furthermore, ammonium also has a slightly higher partition coefficient toward the surface than sodium.^49^ Particularly, in electrospray ionization, the molecules on the surface of the formed droplets are involved in the ionization process,^52,53^ so that one or the other species, the protonated analyte or the corresponding adduct, is preferentially formed. However, this does not explain why complete suppression of sodium adducts is observed only for ammonium acetate or ammonium formate and not for formic acid and also acetic acid.

In addition, Yang et al.^8^ assume that gas-phase reactions are involved to some extent during electrospray ionization. They suggest that solvents or solvent clusters are first protonated or present as sodium adducts. Subsequently, these clusters transfer the charge carrier to the analyte molecule through chemical gas-phase reactions. Therefore, if more [MeOH + Na]^+^ clusters are formed by the addition of sodium acetate, these clusters can ionize the analyte molecules only via their sodium ion. Consequently, if the analyte molecule, as in the case of pyrimethanil, has no corresponding regions for adduct formation with sodium, the suppression of ionization can be explained in this way. This could also be a possible explanation for the suppression of the sodium adduct when ammonium acetate is used, as discussed above. In this case, ammonium solvent cluster form preferentially and then protonate the analyte in the gas phase. Furthermore, our very first experiments with additional CI suggest that adduct formation via gas phase reactions can be suppressed. The fungicide metalaxyl has good preconditions for the formation of sodium adducts compared to the other analytes with a tPSA of 75.5 Å^2^ and four oxygen atoms. Therefore, formation of sodium adducts [M + Na]^+^ can be seen in the pure electrospray ionization mode with *m*/*z* 302. If the plasma source is switched on at a certain time, all sodium adducts are suppressed and only the protonated monomer [M + H]^+^ with *m*/*z* 280 and the well-known transient signals of metalaxyl fragmentation at *m*/*z* 248, 220, and 192 can be observed. The negative ions generated from the plasma source are directed to the sample inlet by the electric field in the desolvation and reaction region, respectively, where they are subsequently discharged. The positive ions are directed toward the drift tube. Along this path, collisions occur with the neutral particles and the generated droplets or ions from the electrospray source. Similar to the APCI, ionization reactions can now occur. As described above, the plasma source provides mainly protonated water clusters, also called reactant ions. These reactant ions can ionize the neutral analyte molecules in the gas phase or they can attach to the generated droplets from the electrospray source and transfer the charge to the droplet. With the charge transferred from the gas phase, protonated solvents or solvent clusters can then preferably form from the solvent droplets, especially since the charge is initially located at the surface. The protonated solvents or solvent clusters can then in turn ionize the analytes, as discussed above. Therefore, with the plasma source active, the ion population can significantly change via gas phase reactions. Nevertheless, a more detailed understanding, especially of the reaction pathways, needs further investigations that will be published in a follow-up paper since the scope of this paper is on adduct formation in ESI/IMS/MS.

However, the addition of 5 mM ammonium acetate prevents the formation of the corresponding sodium adducts. Furthermore, it solves the issue that both ion species, protonated and sodium-bound monomer and beyond that their multimers, have to be observed and thus separated in the IMS if used as a stand-alone device. In addition, trimers are no longer detected in the IMS. Thus, a simple assignment of the resulting peaks in the IMS spectrum becomes possible. This is particularly useful when using an IMS without MS, for example, if the instrumental effort or system size is limited by the application.^54^ This is particularly in line with the 12 basic principles of green chemistry, which is becoming more and more important in general awareness.^55−58^

## Conclusion

This work shows that the addition of additives has a significant influence on the formed ion species in electrospray ionization. Depending on the analyte and the additives selected, the formation of protonated monomers [M + H]^+^ and dimers [2M + H]^+^, the corresponding adducts [M + Na]^+^ and [2M + Na]^+^, or all species occur. In addition, the choice of solvent has an influence on the ion formation and the corresponding predisposition to form more protonated ions or adducts. It was shown that cyprodinil, which should be predominantly protonated, can also exist as a sodium adduct cluster when using acetonitrile as a solvent, or rather as a cluster formation of two analyte molecules, an acetonitrile molecule and sodium ([2M + ACN + Na]^+^, *m*/*z* 513). The acetonitrile here most likely ensures that the topological surface area is increased, so that binding with sodium becomes more likely. For the herbicide isoproturon, the addition of 5 mM sodium acetate leads to the formation of the sodium adduct [M + Na]^+^. In contrast, the addition of 5 mM ammonium acetate leads to the formation of the protonated monomer [M + H]^+^. It was further shown that ionization of the herbicide pyrimethanil via electrospray ionization can be suppressed by the addition of 5 mM sodium acetate, since it does not have appropriate functional groups for the formation of adducts with sodium. Measurements of the mixture of isoproturon and chlortoluron produced a wide variety of ion species and clusters. Accordingly, the assignment in the ion mobility spectrum is difficult. However, the addition of 5 mM ammonium acetate prevents the formation of the corresponding sodium adducts. Thus, it solves the issue that both formed ion species, protonated ions and adducts, have to be observed and thus separated in the IMS if used as a stand-alone device. In addition, trimers are no longer detected in the IMS. Thus, a simple assignment of the resulting peaks in the ion mobility spectrum becomes possible. This is particularly useful when using the IMS without MS, e.g., in field applications, whereas the resolving power of the IMS needs to be further increased to completely separate the protonated ion species of isoproturon and chlortoluron by ion mobility. First experiments with an additional CI during ESI indicate that gas-phase reactions also play a role in the electrospray process and that adduct formation can be suppressed via gas-phase reactions. The use of a plasma-induced additional CI has the advantage that no additives are required and the plasma source can be switched on if required to manipulate ion formation.

## References

[ref1] Cech N. B.; Enke C. G. Practical implications of some recent studies in electrospray ionization fundamentals. Mass Spectrom Rev. 2001, 20, 362–387. 10.1002/mas.10008. 11997944

[ref2] Cech N. B.; Enke C. G. Selectivity in Electrospray Ionization Mass Spectrometry. InCole R. B. (Ed.), Electrospray and MALDI Mass Spectrometry: Fundamentals, Instrumentation, Practicalities, and Biological Applications, 2nd ed.; John Wiley & Sons, Inc.:Hoboken, NJ, 2010; pp 49–73.

[ref3] Bruins A. P. Mechanistic aspects of electrospray ionization. J. Chrom. A 1998, 794, 345–357. 10.1016/S0021-9673(97)01110-2.

[ref4] Van Berkel G. J.; Zhou F.. Electrospray as a Controlled-Current Electrolytic Cell. Anal. Chem. 1995, 67, 3958–3964. 10.1021/ac00117a022.

[ref5] Van Berkel G. J.; Kertesz V. Using the electrochemistry of the electrospray ion source. Anal. Chem. 2007, 79, 5510–5520. 10.1021/ac071944a. 17703524

[ref6] Kebarle P.; Verkerk U. H. Electrospray: from ions in solution to ions in the gas phase, what we know now. Mass Spectrom Rev. 2009, 28, 898–917. 10.1002/mas.20247. 19551695

[ref7] Ehrmann B. M.; Henriksen T.; Cech N. B. Relative importance of basicity in the gas phase and in solution for determining selectivity in electrospray ionization mass spectrometry. J. Am. Soc. Mass Spectrom. 2008, 19, 719–728. 10.1016/j.jasms.2008.01.003. 18325781

[ref8] Yang X. J.; Qu Y.; Yuan Q.; Wan P.; Du Z.; Chen D.; Wong C. Effect of ammonium on liquid- and gas-phase protonation and deprotonation in electrospray ionization mass spectrometry. Analyst 2013, 138, 659–665. 10.1039/C2AN36022E. 23181258

[ref9] Wu C.; Klasmeier J.; Hill H. H. Atmospheric pressure ion mobility spectrometry of protonated and sodiated peptides. Rapid Commun. Mass Spectrom. 1999, 13, 1138–1142. 10.1002/(SICI)1097-0231(19990630)13:12<1138::AID-RCM625>3.0.CO;2-8. 10390859

[ref10] Jemal M.; Hawthorne D. J. Effect of high performance liquid chromatography mobile phase (methanol versus acetonitrile) on the positive and negative ion electrospray response of a compound that contains both an unsaturated lactone and a methyl sulfone group. Rapid Commun. Mass Spectrom. 1999, 13, 61–66. 10.1002/(SICI)1097-0231(19990115)13:1<61::AID-RCM451>3.0.CO;2-2.

[ref11] Asbury G. R.; Hill H. H. Negative Ion Electrospray Ionization Ion Mobility Spectrometry. Int. J. Ion Mobil. Spec. 1999, 2, 1–8.

[ref12] Cole R. B.; Harrata A. Solvent effect on analyte charge state, signal intensity, and stability in negative ion electrospray mass spectrometry; implications for the mechanism of negative ion formation. J. Am. Soc. Mass Spectrom. 1993, 4, 546–556. 10.1016/1044-0305(93)85016-Q. 24227641

[ref13] Scalf M.; Westphall M. S.; Smith L. M. Charge reduction electrospray mass spectrometry. Anal. Chem. 2000, 72, 52–60. 10.1021/ac990878c. 10655634

[ref14] Ebeling D. D.; Westphall M. S.; Scalf M.; Smith L. M. Corona discharge in charge reduction electrospray mass spectrometry. Anal. Chem. 2000, 72, 5158–5161. 10.1021/ac000559h. 11080858

[ref15] Hogan C. J.; Kettleson E. M.; Ramaswami B.; Chen D.-R.; Biswas P. Charge reduced electrospray size spectrometry of mega- and gigadalton complexes: whole viruses and virus fragments. Anal. Chem. 2006, 78, 844–852. 10.1021/ac051571i. 16448059

[ref16] Fernandez de la Mora J. High-Resolution Mobility Analysis of Charge-Reduced Electrosprayed Protein Ions. Anal. Chem. 2015, 87, 3729–3735. 10.1021/ac504445n. 25803189

[ref17] Borsdorf H.; Eiceman G. A. Ion Mobility Spectrometry: Principles and Applications. Appl. Spectrosc. Rev. 2006, 41, 323–375. 10.1080/05704920600663469.

[ref18] Eiceman G. A.; Karpas Z.; Hill H. H. Ion mobility spectrometry, 3rd ed.,; CRC Press: Boca Raton, 2013.

[ref19] Stach J.; Baumbach J. I. Ion Mobility Spectrometry - Basic Elements and Applications. Int. J. Ion Mobil. Spec. 2002, 5, 1–21.

[ref20] Thoben C.; Raddatz C.-R.; Lippmann M.; Salehimoghaddam Z.; Zimmermann S. Electrospray ionization ion mobility spectrometer with new tristate ion gating for improved sensitivity for compounds with lower ion mobility. Talanta 2021, 233, 122579 10.1016/j.talanta.2021.122579. 34215071

[ref21] Hitzemann M.; Schaefer C.; Kirk A. T.; Nitschke A.; Lippmann M.; Zimmermann S. Easy to assemble dielectric barrier discharge plasma ionization source based on printed circuit boards. Anal. Chim. Acta 2023, 1239, 340649 10.1016/j.aca.2022.340649. 36628746

[ref22] Schlottmann F.; Kirk A. T.; Allers M.; Bohnhorst A.; Zimmermann S. High Kinetic Energy Ion Mobility Spectrometry (HiKE-IMS) at 40 mbar. J. Am. Soc. Mass Spectrom. 2020, 31, 1536–1543. 10.1021/jasms.0c00098. 32432872

[ref23] Kwantwi-Barima P.; Reinecke T.; Clowers B. H. Increased ion throughput using tristate ion-gate multiplexing. Analyst 2019, 144, 6660–6670. 10.1039/C9AN01585J. 31595887

[ref24] Chen C.; Tabrizchi M.; Li H. Ion gating in ion mobility spectrometry: Principles and advances. TrAC, Trends Anal. Chem. 2020, 133, 116100 10.1016/j.trac.2020.116100.

[ref25] Allers M.; Timoumi L.; Kirk A. T.; Schlottmann F.; Zimmermann S. Coupling of a High-Resolution Ambient Pressure Drift Tube Ion Mobility Spectrometer to a Commercial Time-of-flight Mass Spectrometer. J. Am. Soc. Mass Spectrom. 2018, 29, 2208–2217. 10.1007/s13361-018-2045-4. 30105740

[ref26] Langejürgen J.; Allers M.; Oermann J.; Kirk A. T.; Zimmermann S. High kinetic energy ion mobility spectrometer: quantitative analysis of gas mixtures with ion mobility spectrometry. Anal. Chem. 2014, 86, 7023–7032. 10.1021/ac5011662. 24937741

[ref27] Kirk A. T.; Zimmermann S. Bradbury-Nielsen vs. Field switching shutters for high resolution drift tube ion mobility spectrometers. Int. J. Ion Mobil. Spec. 2014, 17, 131–137. 10.1007/s12127-014-0153-9.

[ref28] Reinecke T.; Kirk A. T.; Ahrens A.; Raddatz C.-R.; Thoben C.; Zimmermann S. A compact high resolution electrospray ionization ion mobility spectrometer. Talanta 2016, 150, 1–6. 10.1016/j.talanta.2015.12.006. 26838374

[ref29] Bruker Daltonics: micrOTOF II User Manual; Bruker, 2008.

[ref30] Schmidt A.; Bahr U.; Karas M. Influence of pressure in the first pumping stage on analyte desolvation and fragmentation in nano-ESI MS. Anal. Chem. 2001, 73, 6040–6046. 10.1021/ac010451h. 11791577

[ref31] Galaon T.; Vacaresteanu C.; Anghel D.-F.; David V. Simultaneous ESI-APCI+ ionization and fragmentation pathways for nine benzodiazepines and zolpidem using single quadrupole LC-MS. Drug Test. Anal. 2014, 6, 439–450. 10.1002/dta.1526. 23943358

[ref32] Gallagher R. T.; Balogh M. P.; Davey P.; Jackson M. R.; Sinclair I.; Southern L. J. Combined electrospray ionization-atmospheric pressure chemical ionization source for use in high-throughput LC-MS applications. Anal. Chem. 2003, 75, 973–977. 10.1021/ac0205457. 12622394

[ref33] Schappler J.; Nicoli R.; Nguyen D.; Rudaz S.; Veuthey J.-L.; Guillarme D. Coupling ultra high-pressure liquid chromatography with single quadrupole mass spectrometry for the analysis of a complex drug mixture. Talanta 2009, 78, 377–387. 10.1016/j.talanta.2008.11.029. 19203598

[ref34] Brecht D.; Uteschil F.; Schmitz O. J. Development of a fast-switching dual (ESI/APCI) ionization source for liquid chromatography/mass spectrometry. Rapid Commun. Mass Spectrom. 2020, 34, e8845 10.1002/rcm.8845. 32468622

[ref35] Syage J. A.; Hanold K. A.; Lynn T. C.; Horner J. A.; Thakur R. A. Atmospheric pressure photoionization. J. Chrom. A 2004, 1050, 137–149. 10.1016/S0021-9673(04)01362-7. 15508306

[ref36] Cheng S.-C.; Jhang S.-S.; Huang M.-Z.; Shiea J. Simultaneous detection of polar and nonpolar compounds by ambient mass spectrometry with a dual electrospray and atmospheric pressure chemical ionization source. Anal. Chem. 2015, 87, 1743–1748. 10.1021/ac503625m. 25562530

[ref37] Kosarim A. V.; Smirnov B. M.; Capitelli M.; Celiberto R.; Petrella G.; Laricchiuta A. Ionization of excited nitrogen molecules by electron impact. Chem. Phys. Lett. 2005, 414, 215–221. 10.1016/j.cplett.2005.08.012.

[ref38] Michels A.; Tombrink S.; Vautz W.; Miclea M.; Franzke J. Spectroscopic characterization of a microplasma used as ionization source for ion mobility spectrometry. Spectrochimica Acta Part B 2007, 62, 1208–1215. 10.1016/j.sab.2007.08.004.

[ref39] Badal S. P.; Michalak S. D.; Chan G.C.-Y.; You Y.; Shelley J. T. Tunable Ionization Modes of a Flowing Atmospheric-Pressure Afterglow (FAPA) Ambient Ionization Source. Anal. Chem. 2016, 88, 3494–3503. 10.1021/acs.analchem.5b03434. 26916720

[ref40] Chan G.C.-Y.; Shelley J. T.; Wiley J. S.; Engelhard C.; Jackson A. U.; Cooks R. G.; Hieftje G. M. Elucidation of reaction mechanisms responsible for afterglow and reagent-ion formation in the low-temperature plasma probe ambient ionization source. Anal. Chem. 2011, 83, 3675–3686. 10.1021/ac103224x. 21526754

[ref41] da Silva L. A. L.; Sandjo L. P.; Misturini A.; Caramori G. F.; Biavatti M. W. ESI-QTof-MS characterization of hirsutinolide and glaucolide sesquiterpene lactones: Fragmentation mechanisms and differentiation based on Na+ /H+ adducts interactions in complex mixture. J. Mass Spectrom 2019, 54, 915–932. 10.1002/jms.4433. 31476247

[ref42] Matz L. M.; Hill H. H. Evaluation of opiate separation by high-resolution electrospray ionization-ion mobility spectrometry/mass spectrometry. Anal. Chem. 2001, 73, 1664–1669. 10.1021/ac001147b. 11338577

[ref43] Clowers B. H.; Steiner W. E.; Dion H. M.; Matz L. M.; Tam M.; Tarver E. E.; Hill H. H. Evaluation of sulfonylurea herbicides using high resolution electrospray ionization ion mobility quadrupole mass spectrometry. Field Analyt. Chem. Technol. 2001, 5, 302–312. 10.1002/fact.10010.

[ref44] Sugimura N.; Furuya A.; Yatsu T.; Shibue T. Prediction of adducts on positive mode electrospray ionization mass spectrometry: proton/sodium selectivity in methanol solutions. Eur. J. Mass Spectrom. 2015, 21, 725–731. 10.1255/ejms.1389. 26579928

[ref45] Mortier K. A.; Zhang G.-F.; van Peteghem C. H.; Lambert W. E. Adduct formation in quantitative bioanalysis: effect of ionization conditions on paclitaxel. J. Am. Soc. Mass Spectrom. 2004, 15, 585–592. 10.1016/j.jasms.2003.12.013. 15047063

[ref46] Kruve A.; Kaupmees K.; Liigand J.; Oss M.; Leito I. Sodium adduct formation efficiency in ESI source. J. Mass Spectrom 2013, 48, 695–702. 10.1002/jms.3218. 23722960

[ref47] Kruve A.; Kaupmees K. Adduct Formation in ESI/MS by Mobile Phase Additives. J. Am. Soc. Mass Spectrom. 2017, 28, 887–894. 10.1007/s13361-017-1626-y. 28299714

[ref48] Rebane R.; Kruve A.; Liigand J.; Liigand P.; Gornischeff A.; Leito I. Ionization efficiency ladders as tools for choosing ionization mode and solvent in liquid chromatography/mass spectrometry. Rapid Commun. Mass Spectrom. 2019, 33, 1834–1843. 10.1002/rcm.8545. 31381213

[ref49] Pegram L. M.; Record M. T. Quantifying accumulation or exclusion of H+, HO-, and Hofmeister salt ions near interfaces. Chem. Phys. Lett. 2008, 467, 1–8. 10.1016/j.cplett.2008.10.090. 23750042 PMC3673785

[ref50] Tse Y.-L. S.; Chen C.; Lindberg G. E.; Kumar R.; Voth G. A. Propensity of Hydrated Excess Protons and Hydroxide Anions for the Air-Water Interface. J. Am. Chem. Soc. 2015, 137, 12610–12616. 10.1021/jacs.5b07232. 26366480

[ref51] Li Z.; Li C.; Wang Z.; Voth G. A. What Coordinate Best Describes the Affinity of the Hydrated Excess Proton for the Air-Water Interface?. J. Phys. Chem. B 2020, 124, 5039–5046. 10.1021/acs.jpcb.0c03288. 32426982

[ref52] Cech N. B.; Enke C. G. Relating electrospray ionization response to nonpolar character of small peptides. Anal. Chem. 2000, 72, 2717–2723. 10.1021/ac9914869. 10905298

[ref53] Cech N. B.; Enke C. G. Effect of affinity for droplet surfaces on the fraction of analyte molecules charged during electrospray droplet fission. Anal. Chem. 2001, 73, 4632–4639. 10.1021/ac001267j. 11605841

[ref54] Thoben C.; Werres T.; Henning I.; Simon P. R.; Zimmermann S.; Schmidt T. C.; Teutenberg T. Towards a miniaturized on-site nano-high performance liquid chromatography electrospray ionization ion mobility spectrometer with online enrichment. Green Anal. Chem. 2022, 1, 100011 10.1016/j.greeac.2022.100011.

[ref55] Pawliszyn J.; Barceló D.; Arduini F.; Mondello L.; Ouyang Z.; Nowak P. M.; Wietecha-Posłuszny R. Green analytical chemistry-a new Elsevier’s journal facing the realities of modern analytical chemistry and more sustainable future. Green Anal. Chem. 2022, 1, 100001 10.1016/j.greeac.2022.100001.

[ref56] Armenta S.; Garrigues S.; La Guardia M. de Green Analytical Chemistry. TrAC Trends Anal. Chem. 2008, 27, 497–511. 10.1016/j.trac.2008.05.003.

[ref57] Koel M.; Kaljurand M. Application of the principles of green chemistry in analytical chemistry. Pure Appl. Chem. 2006, 78, 1993–2002. 10.1351/pac200678111993.

[ref58] Pallone J. A. L.; Caramês E.T.d.S.; Alamar P. D. Green analytical chemistry applied in food analysis: alternative techniques. Curr. Opin. Food Sci. 2018, 22, 115–121. 10.1016/j.cofs.2018.01.009.

